# Patterns of Horse-Rider Coordination during Endurance Race: A Dynamical System Approach

**DOI:** 10.1371/journal.pone.0071804

**Published:** 2013-08-05

**Authors:** Sylvain Viry, Rita Sleimen-Malkoun, Jean-Jacques Temprado, Jean-Philippe Frances, Eric Berton, Michel Laurent, Caroline Nicol

**Affiliations:** 1 Aix-Marseille Université, CNRS, Institut des Sciences du Mouvement, Marseille, France; 2 BRD Concept, Anglet, France; 3 Ecuries JPF, Venelles, France; The University of Queensland, Australia

## Abstract

In riding, most biomechanical studies have focused on the description of the horse locomotion in unridden condition. In this study, we draw the prospect of how the basic principles established in inter-personal coordination by the theory of *Coordination Dynamics* may provide a conceptual and methodological framework for understanding the horse-rider coupling. The recent development of mobile technologies allows combined horse and rider recordings during long lasting natural events such as endurance races. Six international horse-rider dyads were thus recorded during a 120 km race by using two tri-axial accelerometers placed on the horses and riders, respectively. The analysis concentrated on their combined vertical displacements. The obtained shapes and angles of Lissajous plots together with values of relative phase between horse and rider displacements at lower reversal point allowed us to characterize four coordination patterns, reflecting the use of two riding techniques per horse's gait (trot and canter). The present study shows that the concepts, methods and tools of self-organizing dynamic system approach offer new directions for understanding horse-rider coordination. The identification of the horse-rider coupling patterns constitutes a firm basis to further study the coalition of multiple constraints that determine their emergence and their dynamics in endurance race.

## Introduction

During the last 30 years, the growing interest in horse racing and riding activities has stimulated scientific research in biomechanics and physiology of equine locomotion (see [1], for an overview). However, studies predominantly focused either on the horse or on the rider, considered as separate systems, to describe the fatigue induced via prolonged riding. Conversely, the complex interactions between the horse and the rider were scarcely explored (see [2], [3], [4], [5], for noticeable exceptions). In addition, horse-rider interactions were only investigated in experimental situations of short duration and during prescribed horse gait and riding techniques (see [5] for an illustrative example). Thus, the motivation of the present study was to investigate horse-rider interactions in a natural, long duration situation of endurance race, since these interactions may significantly affect the preservation of functional status of the horse, which is a primary goal for the rider in endurance race [6]. Unfortunately, although horse-rider interactions have recently been considered as a desirable objective in the literature [2], [4], [7], [8], they remain poorly understood. A possible discouraging constraint and a limiting factor is that the analysis of horse-rider interactions in natural situations of endurance races requires a set of sophisticated motion analysis equipment (e.g. [8]). In the present paper, we contend that accelerometer measurement tools might be a simple method of analysis to characterize the coordination patterns between the horse and the rider during the race. To achieve this objective, we used motion information recorded cycle-by-cycle over the entire duration of the race, through specific tri-axial (3D) accelerometer measurement systems usually dedicated to gait patterns analysis in human and horse (Locometrix® and Equimetrix®, respectively). Previous studies examining horse's gait during successive strides have demonstrated the higher repeatability of the acceleration shape along the vertical (dorsoventral) as compared to the longitudinal (antero-posterior) axis; the latter one being more influenced by the propelling work of each limb cycle [9], [10]. Based on these observations, the present analysis of the horse and rider interactions concentrated on their displacements along the vertical axis: dorsoventral for the horse *vs.* craniocaudal for the rider. In a recent paper [5], Wolframm and collaborators used similar measurement tools to study horse gaits and riding techniques during a dressage race that is, walk, rising trot, sitting trot and sitting canter. They demonstrated that in these controlled situations of short durations (gait and techniques were imposed to horse-rider dyads over few loops of 20 meters), accelerometer devices were appropriate to record simple kinematic variables, which allowed describing horse-rider coordination patterns. The present study aims to extend the use of accelerometer device to a more natural (i.e. uncontrolled and long duration) situation of endurance race, in which patterns of horse-rider coordination are not imposed by experimenters and, hence can only be detected from blind analysis of raw data.

Theoretically, the approach of horse-rider interactions adopted in the present study is inspired from the framework of Coordination Dynamics (CD) that is, the science of coordination in biological systems introduced almost 30 years ago by Kelso and collaborators [11], [12], [13], [14], [15], [16]. Grounded on Synergetics [11], a theory of self-organization in complex systems, this approach has provided new concepts and tools for understanding the mechanisms and principles underlying the emergence, stabilization, destabilization and changes of coordination patterns in a wide range of biological, chemical and physical systems [11], [15], [16]. The basic assumption of CD is that, whatever the nonlinear complex system under consideration, coordination patterns arise spontaneously as the result of self-organization, from the mutual coupling among interacting subsystems (e.g., neural, muscular, mechanical, energetic, environmental). As a corollary, a key assumption is that coordination dynamics deals with informational quantities of a relational kind that couple the different parts of the system or the different sub-systems. Consequently, patterns of coordination can be characterized by a low dimensional collective variable (so-called order parameter, [11]). On the basis of order parameter analysis, pattern stability, loss of stability and the eventual transitions between existing patterns can be captured. These phenomena manifest the existence of the underlying coordination dynamics of the system (see [15], [16] for overviews and details).

CD has been primarily applied to the study of inter-limb coordination in both human [13], [14], [17] and animal beings (i.e., horse gait, see [18], [19], [20]). In this context, inter-limb coordination is considered as the result of emergent dynamics in a system of weakly coupled oscillators linked through a nonlinear informational coupling. Accordingly, relative phase is commonly used as the order parameter for capturing coordination patterns between limbs (see [15] for overviews in both bimanual and quadrupedal coordination tasks). Indeed, relative phase quantify the spatio-temporal relationship between the two interacting components independent of movement amplitude and oscillation frequency of the limb components. Consequently, variability of relative phase is frequently used as a complementary measure to assess the stability of identified coordination patterns.

In addition to inter-limb coordination tasks, one of the most promising extensions of the concepts, methods and tools of informationally coupled self-organizing systems has been shown in inter-personal coordination tasks, from synchronized clapping in large audiences ([21], [22]) to intentional sensorimotor coordination [23], [24], see [25] for an overview). In this perspective, CD allowed the exploration of the mechanisms mediating the emergence and dissolution of bonds between individuals by quantifying real time inter-agent coupling processes. Schmidt et al.'s study [23], can be considered as the seminal and paradigmatic exploration of inter-personal coordination. Its contribution of [23] was to univocally demonstrate that coordination phenomena found within a person's brain or body could be extended to the interactions between people. In this study, two individuals sitting next to each other were asked to swing their legs adopting either an in-phase or anti-phase pattern (i.e., moving in the same or opposite directions). As movement frequency was increased, predicted features of phase transitions in self-organizing systems were observed: i) the two coordination patterns were differentially stable, (ii) phase transitions occurred from the less stable coordination pattern (anti-phase) to the more stable one (in-phase); (iii) relative phase variability dramatically increased before transition.

Based on the framework of CD, it can be assumed that phase synchronization between the horse and the rider is a special case of inter-personal coordination that belongs to a family of processes generic to the organization of complex brain-behavior systems [15], [26]. Indeed, in horse riding, the horse and the rider both move up and down through translational motion as a result of mechanical constraints associated to the horse's gait, but the rider must continuously adapt his/her movement to assemble a coordination pattern that: i) optimizes efficiency [4] and ii) permits to ensure horse control through informational contact like rider–saddle contact for instance. Supporting evidence of information coupling in the (complex) horse-rider system has been provided in previous studies. For instance, Lagarde et al. [2] showed that stability of synchronization between rider and horse motions was increased in expert riders, relative to novices. In addition, Peham et al. [27] have shown that a skilled rider is able to stabilize a horse trotting on a treadmill. To account for the dynamics resulting from intentional forcing of horse-rider coupling, Lagarde et al. [2] suggested that the contact between the horse and the rider (through the saddle) might convey haptic information that is functional for driving horse gait.

These findings convinced us that one could capitalize on the basic principles established by CD in inter-personal coordination to provide a conceptual and methodological framework for understanding horse-rider interactions during endurance race. Accordingly, in the present study, we aimed to provide a method to characterize the dynamics of the horse-rider system using simple and unified representations of macroscopic variables capturing the horse-rider coupling (HRC) in the various contextual situations of an endurance race. Specifically, by analyzing the respective displacements of the rider and the horse along the vertical axis during either different horse gaits (trot *vs.* canter) or riding techniques (sitting, rising and two-point), we aimed to characterize coordination patterns emerging in endurance race.

Our first, general hypothesis was that the combination of horse gait and adopted riding technique should manifest in a limited number of specific horse-rider coordination patterns. Specifically, on the one hand, one should observe particular coordination patterns per horse gait, corresponding to each of the different adopted techniques. On the other hand, since the two-point technique can be adopted at both trot and canter, we predicted to observe comparable horse-rider coordination patterns for these combinations. It would suggest that coordination patterns are more strongly constrained by rider technique than by horse gait. Another general hypothesis was that, at lower reversal point of the cycle, stability of some horse-rider coordination patterns should be improved by the use of haptic/tactile information associated to the rider/saddle contact. It should lead the rider to be more tightly coupled with horse's displacements. Thus, at least during the sitting technique, we expected to observe: 1) a lower value of relative phase at reversal point of the horse displacement cycle, and 2) lower variability of relative phase, relative to other gait-technique combinations.

## Methods

### Rider and horse

6 expert horse-rider dyads, which had at least 3 international podiums within the last 5 years, have been successfully recorded in international endurance races at free speed (minimum of 12 km.h^−1^) over 120 to 139 km in a day. The group of riders included three females and three males (mean age = 29±3 years; body mass = 60±9 kg; height = 1.71±0.09 m), three gelding horses and three mares (age = 9±1 years; body mass = 364±57 kg; height at the withers = 1.56±0.04 m). All riders and horses were competing at national and international levels for more than 5 years. No instruction was given to the rider concerning the specific techniques to adopt during the race. This procedure favored spontaneous patterns of coordination to accommodate current race constraints. Before their enrollment in the study, all subjects were fully informed of the entire protocol and signed the consent form. This study was approved by the ethics committee of Aix-Marseille University and it conforms to the provisions of the Declaration of Helsinki.

### Racing conditions

Data were recorded during 6 different endurance races (2012, April to October). The races were classified as International Equestrian competitions of around 130 km to be covered in a day, with positive/negative changes varying from ±504 m to ±3344 m. Each race was subdivided in 4 loops (L1–L4) varying from 20 to 42 km. They included roads and forest track conditions. Veterinary inspections ‘Vet Gates’ took place before and after the race as well as between the loops. To be ranked, the horse had to pass in each of these inspections.

### Data acquisition

Two tri-axial (3D) accelerometer data loggers – Locometrix®, (henceforth Locom, INRA, SGQA, France) and Equimetrix® (henceforth Equim, Centaure Metrix, France) – were used to record rider and horse accelerations, respectively (full scale of measurements: Locom = ±6 g; Equim = ±10 g; sensitivity = 16.4 mV/g; resonance frequency = 550 Hz). Conforming to the recommended instructions for their utilization, the accelerometers were rigidly fixed on rider and horse bodies as follows: Locom was fixed onto a neoprene kidney belt worn by the rider, and Equim was secured inside a modified girth strap of the saddle and adjusted under the caudal part of the sternum [9]. In these locations, the accelerometers recorded the vertical (dorsoventral for the horse *vs.* craniocaudal for the rider), the antero-posterior (fore-aft) and mediolateral (side to side) accelerations of the horse and rider. Sampling rate was 100 Hz per axis with an anti-aliasing filter (cut-off frequency of 50 Hz). To allow subsequent synchronization of the rider and horse accelerometric data, both Locom and Equim accelometers were hit together on the vertical axis before and after completion of each loop. The data were then transferred to a laptop computer for further analyses.

### Accelerometric data processing and analysis along the vertical axis

The synchronized 3D acceleration signals of the six horses and riders were processed for each stride (defined as a full cycle of limb motion) by signal analysis procedures developed under a scientific software environment (Matlab 7). Their respective 3D displacements were obtained by double integration using cumulative trapezoidal numerical integration. A Butterworth bandpass filter (1–12 Hz) was used to remove the drift (low frequency) created by the double integration as well as any perturbation associated to a brisk movement. This filter was applied with a zero-time lag Butherworth filter to keep the signal without any phase shift.

The analysis of the horse-rider interactions concentrated on their displacements along their vertical axis: dorsoventral (DV) for the horse *vs.* craniocaudal (CC) for the rider. This choice was based on the previously reported high consistence of the horse's DV acceleration shape [9], [10]. This allowed also comparison to previous studies on the dynamics of horse-rider system along the vertical axis [2], [27].

**Figure 1:**
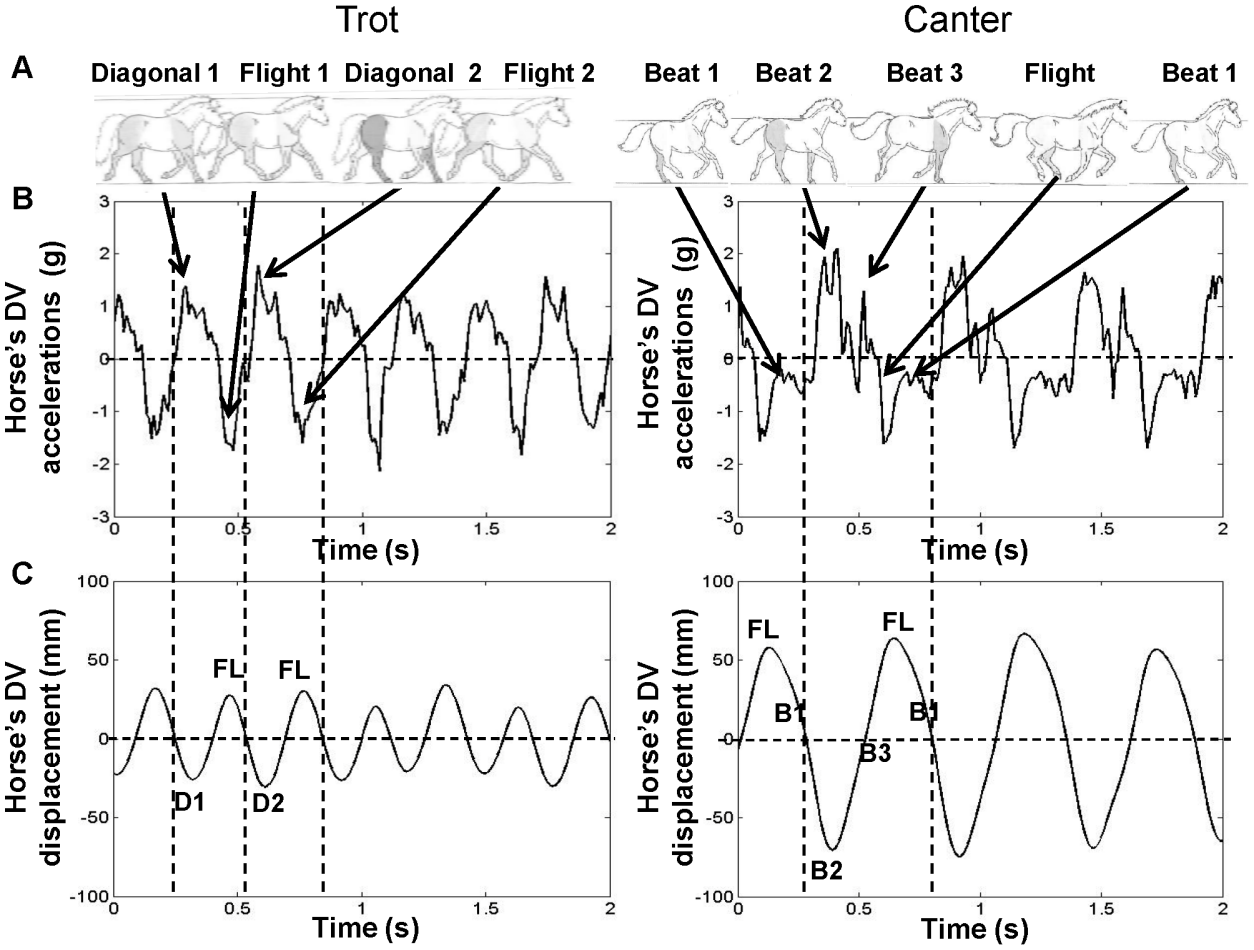
Accelerometer data analysis along the vertical axis at the trot and canter. Panel A: Representative coordination patterns between horse's limbs for each gait. Panel B: Recorded dorsoventral (DV) acceleration signal. Panel C: Calculated DV displacement. As shown by the vertical dashed lines, the DV displacement presents two repeated oscillations per stride at the trot (2 contact and flight phases) and only one at the canter. The corresponding DV frequency at the trot is thus higher than at the canter. The description of the acceleration data is based on the previous works of Barrey et al. [1], [10] at the trot and canter, respectively.

As usually done with accelerometric data analyses [28], [29], the mean signal was subtracted in order to center the displacement on a zero value. Detailed patterns of the links between the horse limb patterns, the recorded dorsoventral (DV) acceleration signal and the calculated dorsoventral displacement are presented for the three strides of one horse at trot and canter in Figure 1.

#### Identification of horse's gait patterns

Horse gait patterns (walk, trot and gallop) can be indirectly but unequivocally, identified by the range of stride frequency at which they appear [30]. Specifically, although stride frequencies are too close to each other at trot and canter (slow gallop) to allow differentiation of horse's gait pattern, this can be obtained on the basis of the analysis of the corresponding DV displacement. As shown in Figure 1, DV displacement presents two repeated oscillations per stride at the trot (2 contact and flight phases) *vs.* one at the canter. As shown by Barrey et al. [1], [9], [10], [31], the DV displacement frequency corresponds indeed to the half stride at the trot and to the stride at the canter. Differentiation of the horse's gait patterns was thus inferred in the present study from the period of horse's DV oscillation, which allowed counting the number of strides performed at the trot and at the canter.

As a proof-of-concept and methodological validation, the method was developed with one expert horse-rider dyad and then applied to the other 5 dyads. To limit the presumed influence of both stress and strategy on the performance in the first loop, as well as of fatigue and strategy on the last one, the analysis concentrated on the two intermediate loops (L2 and L3) of each race.

Once the number of strides to be considered in each horse gait was selected, we applied a second criterion to infer the techniques adopted by the rider in each horse gait.

#### Identification of riding techniques per horse's gait

In endurance races, it has been observed that two major riding techniques are commonly used for each horse gait [32]. The so-called “two-point technique” is currently used in both trot and canter, thereby leading to the *two-point trot and two-point canter* techniques, respectively. Irrespective of whether the two-point technique is used in trot or canter, the rider remains off the saddle that is, in equilibrium in his two stirrups, with only the rider's calves contacting the horse. Another currently used technique is the so-called “*rising trot*”. It consists for the rider of making an up and down movement per stride, rising out from the saddle for one beat (half stride) and going down to touch lightly the saddle on the second one. Finally, in the *sitting canter* technique, the rider remains mostly in contact with the saddle, making a sweeping motion with the hips. We expect that other gaits and techniques described in [7], such as walk and sitting trot, are very occasionally used in endurance race. The identification of the riding techniques adopted at the trot and canter was obtained by quantifying the amplitude of the rider cranio-caudal displacement. In Pfau et al. study [4], the use of the two-point technique in gallop was associated with small amplitude of vertical rider's displacements in the reference frame inertial world. The authors interpreted this observation as the result of compensation for horse's DV displacement by the rider. Thus, in each horse gait (i.e., trot and canter), we have calculated the distribution of rider's amplitude of CC displacement. A method of minimization of error threshold was used to determine the optimal threshold to differentiate the distributions obtained in each gait (see [33] for a detailed description). Such distributions allowed us distinguishing two groups of strides in each gait, which presumably corresponded to different rider's techniques. We inferred that the strides associated with the smaller amplitude corresponded to the two-point technique. Then, we were able to unambiguously distinguish the two-point trot and two-point canter from the other techniques. For the two remaining groups of strides, we easily identified those corresponding to the sitting canter technique since they were mechanically associated with the larger rider's displacements due to the prolonged contact with the saddle (see [5] for a consistent description). Consequently, the other group of strides identified at the trot necessarily corresponded to the rising technique, and this was confirmed by the specificity of the rider DV displacement shape as described in [5].

#### Identification of HRC patterns

**Figure 2:**
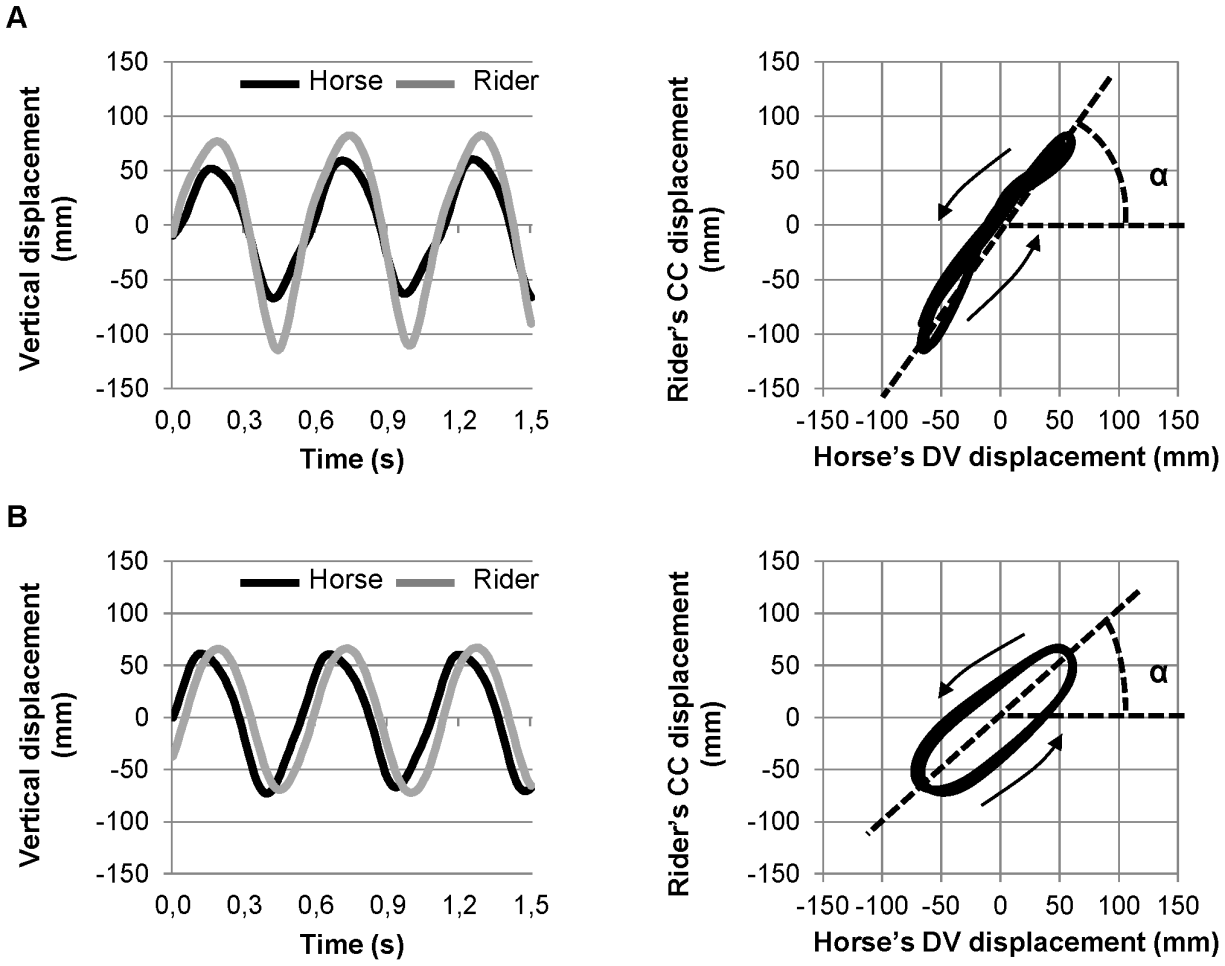
Lissajous plots of horse (x axis) and rider's (y axis) vertical displacements. A angle reflects the ratio between the amplitude of the horse's dorsoventral (DV) and rider's craniocaudal (CC) displacements. A reduction of the rider's CC displacement, while horse's DV displacement was kept constant, resulted in a smaller α angle (panel B vs. A). The global form of the Lissajous plot illustrates difference in spatiotemporal relationship (relative phase) between the horse and the rider along the stride cycle. The sign of the relative phase is related to the sense of rotation of the plot indicated by the small arrows (counter-clockwise for a positive delay when the rider's reversal movement is delayed as compared to the horse's one).

To capture the horse-rider dynamics that is, the existence of specific and different coordination patterns between the horse and the rider, their spatio-temporal relationship over stride cycles were represented on a displacement/displacement space (Lissajous plots). This method has been commonly used in inter-limb coordination studies (e.g., [34]) to provide a simplified picture of the spatiotemporal relationship between limb motions corresponding to different coordination patterns (e.g., [35], [36], [37]). In the present study, the overall differentiation of gait patterns and riding techniques was based on analyzing the horse and rider displacements along the vertical axis (DV: dorsoventral and CC: craniocaudal displacements, respectively) so that the Lissajous plots corresponded to spatio-temporal relationship in this direction (Figure 2). For each Lissajous plot, the horse's DV and rider's CC displacements per stride were time normalized and combined with each other. To allow subsequent differentiation of the riding techniques within each gait, these data were not normalized in amplitude. Since the spatial coordinates of the horse and rider were unknown in Galilean frame, their displacements were centered on their respective average position.

As illustrated in Figure 2, Lissajous plots (i.e., displacement-displacement plots) were used to characterize the horse-rider spatio-temporal relationship during each stride. The different patterns of horse-rider coordination were identified according to classic profiles of Lissajous plots. Lissajous profiles close to oblique line (45° to the right) reflected quite perfect in-phase vertical horse and rider's displacements along the stride (panel A). Deviations from perfect line were observed in case of a constant time delay in the spatiotemporal relationship, thereby leading to a rounder shape (panel B).

In addition to global profiles of Lissajous plots, the horse-rider spatio-temporal relationship was quantified at specific points of the stride cycle by calculating the relative phase (RP), which is the phase difference between the rider and the horse in their cycle. Since horse and rider's oscillatory displacements were not perfectly harmonic, RP was calculated at lower reversal point of the displacement using the discrete point estimate method (see [38], [39] for an overview of calculation methods). The time delay that separated the rider from the horse at their respective lower point of vertical displacement was measured for each stride and transformed into relative phase (RP) by applying the classic formula used for calculation of discrete RP [40]:
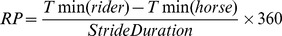



It is based on the time difference between the occurrence of minimal displacement of the rider and that of the horse closest in time was first calculated. This duration was expressed in degrees relative to the period of the horse stride.

When the horse and rider displacements were in phase at this point, the RP was close to zero (Figure 2, panel A). A negative RP was obtained when the rider was ahead of the horse and a positive RP in the opposite situation. As illustrated in Figure 2 (panel B), a positive RP was associated with a counter-clock wise sense of rotation of the Lissajous plot. The HRC corresponding to each of the two riding techniques per horse's gait was thus characterized by its mean RP and standard deviations.

As illustrated also in Figure 2, the vertical horse and rider's displacements resulted in a Lissajous plot with a given orientation (α angle). For a similar horse's DV displacement, any reduction in the amplitude of the rider's CC displacement resulted in a decrease in α angle (Figure 2, panel B *vs.* A). Thus, this parameter was used to investigate the difference in coordination patterns between the two riding techniques within each type of gait. The method of minimization of error threshold (previously used to differentiate the riding techniques) was then applied to the bimodal stride distribution obtained for each gait.

### Statistical analysis

Separate between subjects one-way ANOVAs were carried out on the mean values of each dyads in the 4 gait-technique combinations (within subject) to compare: 1) α angles for the Lissajous' plots corresponding to the different patterns, 2) mean RP at the lower reversal point of the cycle and 3) Standard Deviation of RP. Newman-Keuls *Post-hoc* tests were used to identify the significant differences between patterns. All data were analyzed using dedicated software package (STATISTICA, version 10, stat-Soft, Inc. Tulsa, Oklaoma, USA). In all tests and comparisons, *p*-values less than 0.05 were considered significant.

## Results

Firstly, as a proof-of-concept and methodological validation, a detailed analysis is presented for one representative horse-rider dyad – a female rider (age = 27 years; body mass = 50 kg; height = 1.63 m) and a gelding horse (age = 10 years; body mass = 320 kg; height at the withers = 1.50 m). Then, the analysis is extended to the 5 other dyads. Mean Lissajous' plots are presented. Individual patterns are available as supplementary material (Figures S1, S2, S3, and S4).

### Individual analysis of one dyad

The first analyzed horse-rider dyad (D1) won the endurance race that included 53 competitors, by riding at a mean speed of 18.07 km.h^−1^ (5.02 m.s^−1^) and passing all the vet gates (see FEI Rules for Endurance Riding at: http://www.fei.org/disciplines/endurance/rules). L2 and L3 loops were performed at averaged speeds of 5.14 and 4.31 m.s^−1^, respectively.

#### Identification of the horse's gait patterns in D1

To distinguish between strides performed at canter and trot, we analyzed the frequency of the DV horse displacement. Due to the different nature of inter-limb coordination patterns adopted at the trot and canter, higher DV displacement frequencies were expected to be observed in the former horse's gait. Accordingly, mean DV oscillation frequencies observed for strides performed at the trot and canter were 3.19±0.25 Hz and 1.85±0.17 Hz, respectively. For the trot, it corresponded to a stride frequency of 1.60±0.07 Hz.

Then, we calculated the relative proportion of trot and canter in the number of strides recorded. This analysis revealed that trot was more frequently used than canter (L2: 63 *vs.* 37% and L3: 71 *vs.* 29%). It corresponded to a total duration of 118 min at the trot and 54 min at the canter.

#### Identification of the riding techniques per horse's gait in D1

The subsequent analysis of the maximal amplitude of the rider's craniocaudal (CC) displacement per stride showed that at both trot and canter, the distribution of rider's CC displacement was bimodal. For each group of strides, the lower range of displacement was attributed to the two-point technique.

**Figure 3:**
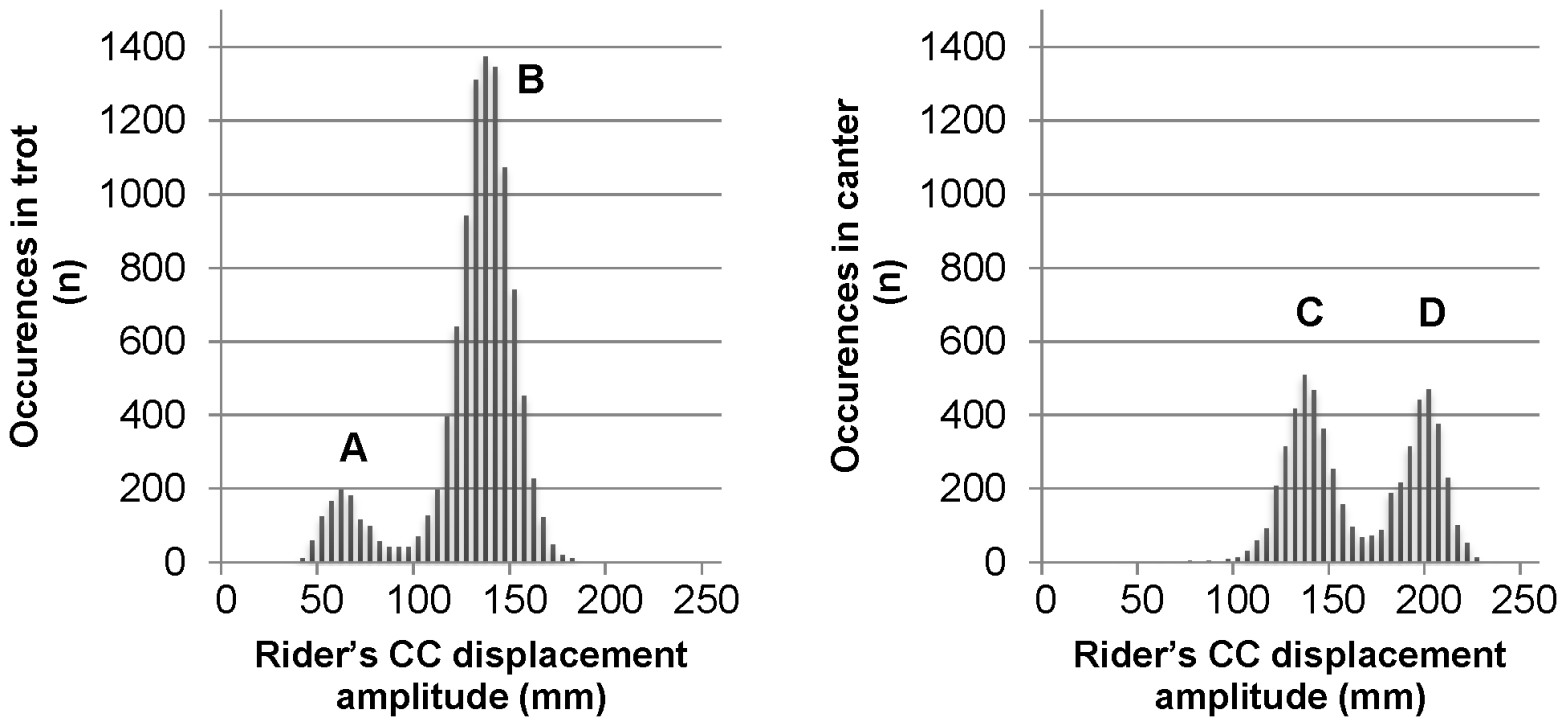
Quantification of the stride occurrences for each of the two riding techniques per horse's gait. The stride distribution at the trot (left graph) and at the canter (right graph) is based on the rider's craniocaudal (CC) displacement, which is of lower amplitude in the two-point technique (A and C) as compared to its value in either sitting canter (D) or rising trot (B). The latter technique was mostly used by the present horse-rider system (dyad 1) in the two loops under interest.

As shown in Figure 3 for the strides performed at the trot during the two intermediate loops, the rider's CC displacement averaged 63.7±13.0 in the two-point trot technique (A) and 131.4±14.2 mm in the rising technique (B). During the strides performed at the canter, amplitude was 134.7±17.3 mm in two-point technique (C) and 194.6±11.2 mm in the sitting technique (D). As expected, amplitude observed during sitting canter was larger than in the other techniques. The similar range of values observed in the rising trot and in the two-point canter techniques confirms the necessity of a preliminary gait differentiation.

Subsequent analysis of strides performed at the trot revealed a major use (89%) of the rising trot technique whereas the two-point trot represented only 11%. In strides performed at the canter, the percentages of the two-point and sitting canter techniques were balanced (53 and 47%, respectively).

#### Coordination patterns between the horse and the rider (HRC) in D1

**Figure 4:**
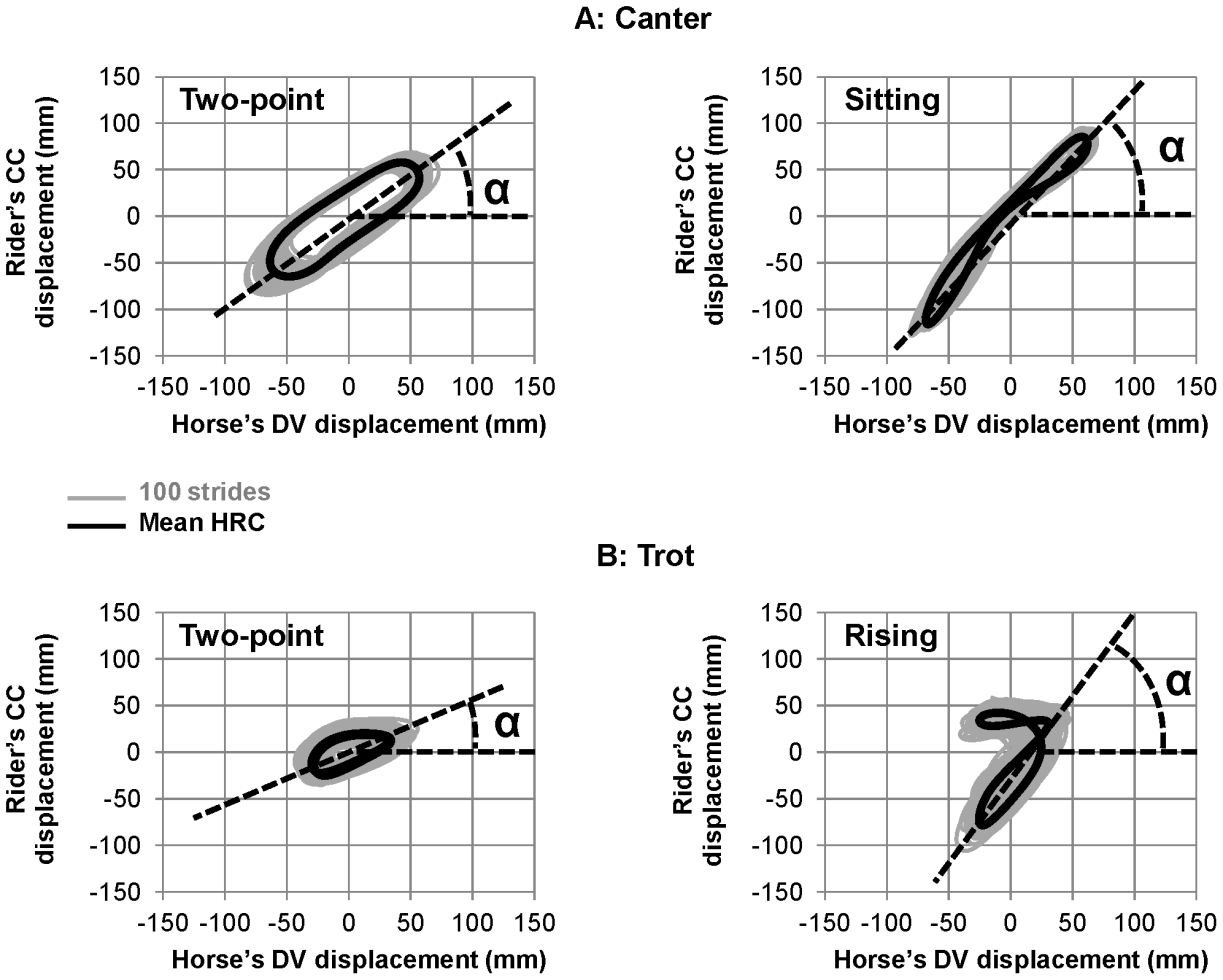
Influence of the gait pattern and riding technique on the horse-rider coupling (HRC). Each Lissajous plot combines the horse's dorsoventral (DV) displacement (horizontal axis) with the rider's craniocaudal (CC) one (vertical axis) for each horse's gait (panel A *vs.* B) and riding technique (left *vs.* right panels). The shape of the Lissajous plot is clearly gait specific: the trot (panel B) is characterized by two successive coordination patterns instead of one at the canter (panel A), which may either overlap (two-point technique) or differ from each other (rising trot). At both trot and canter differentiation of the riding technique can be made based on the lower inclination (α angle) of the Lissajous plot in between its two points of reversal. Emphasizing the stability of the HRC patterns, the mean Lissajous plot (black line) is very similar to the plots of 100 successive strides (grey line).

As expected, Lissajous plots revealed different types of horse-rider coordination patterns for each association of horse gaits and riding techniques (Figure 4).

The fact that each horse's stride includes two DV oscillations at the trot and only one at the canter could be identified in the Lissajous plots that reflect HRC. It is noticeable that the mean Lissajous plots obtained for the two loops was very similar to the plots obtained for 100 successive strides (Figure 4).

**Figure 5:**
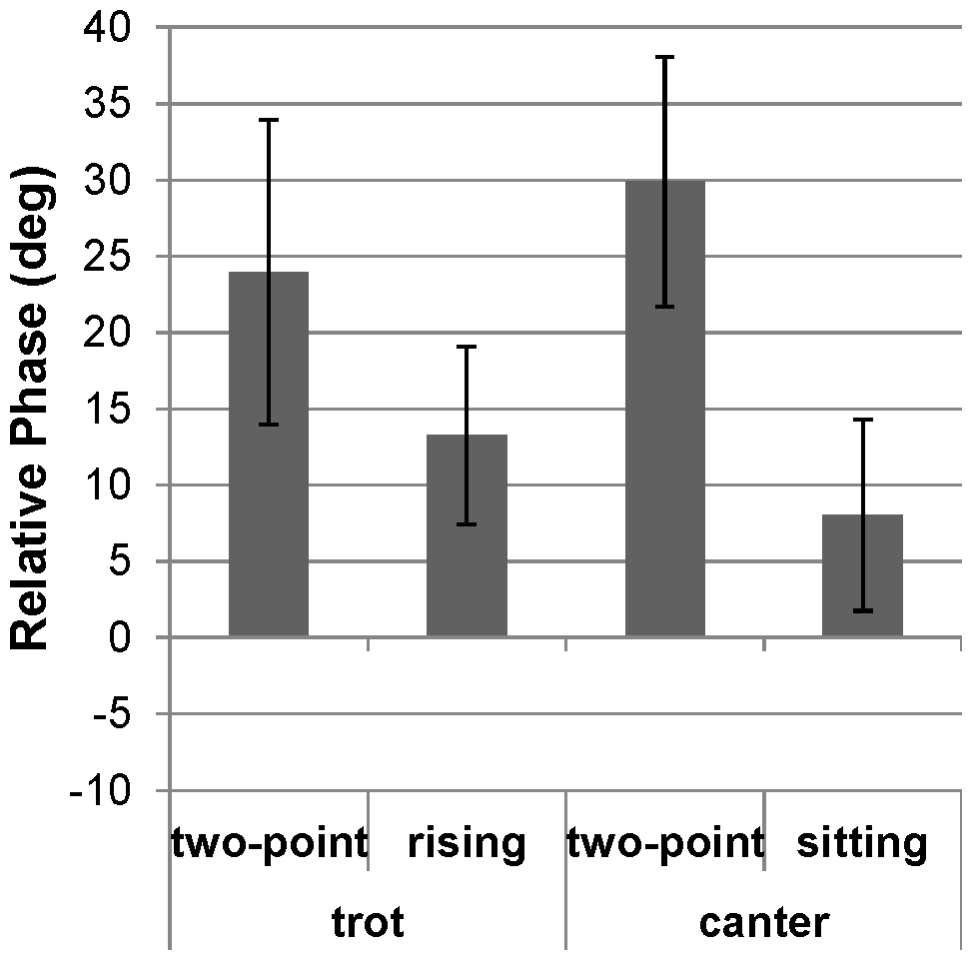
Influence of horse's gaits and riding techniques on relative phase at the lowest reversal point. Mean and standard deviations of the relative phase at the lowest point of the horse's and the rider's vertical displacements in each riding gait and technique. A positive RP value means that the horse reaches the lowest point before the rider. The higher RP values in the two-point techniques indicate a greater delay than in the two other techniques (rising trot and sitting canter).

**Figure 6:**
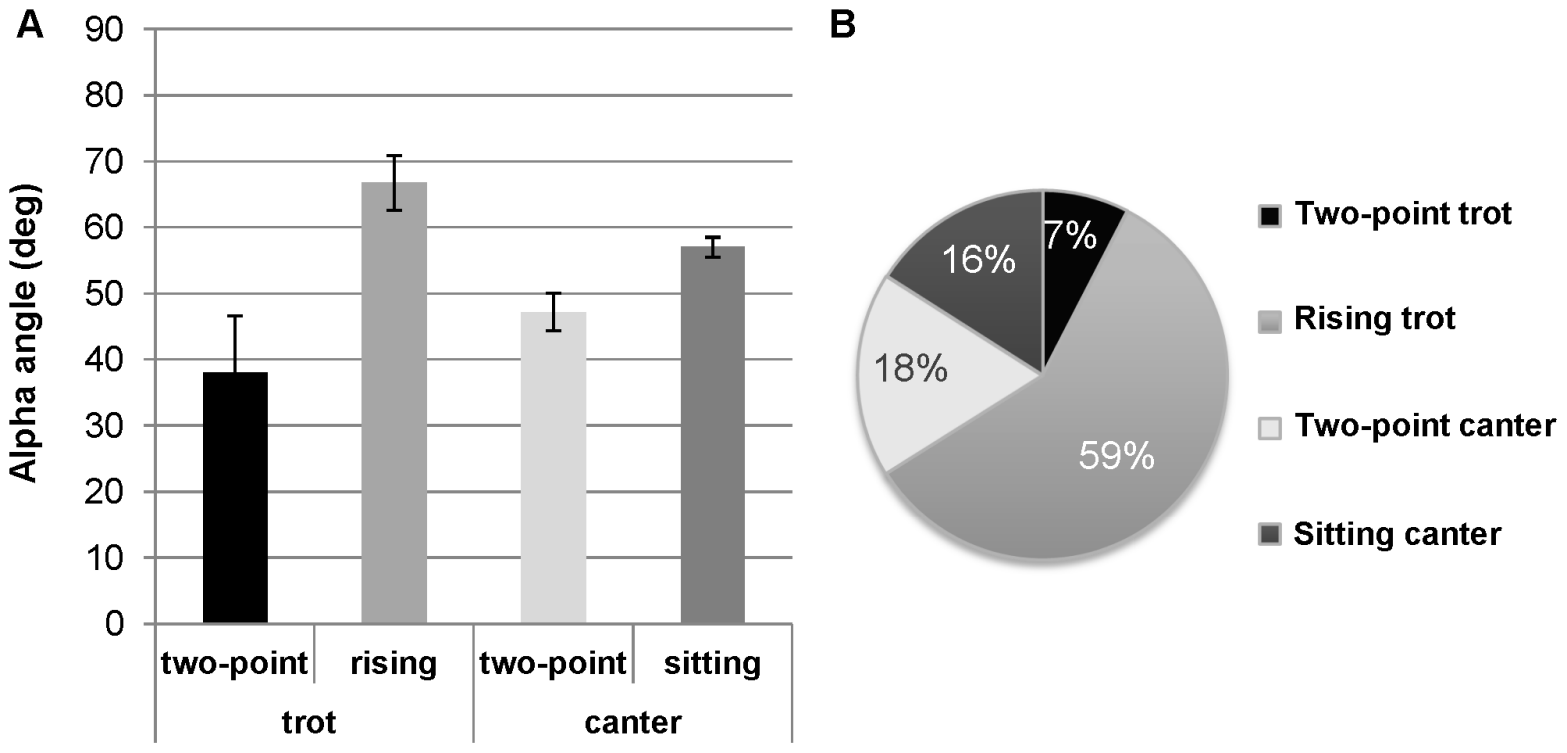
Differentiation of horse's gaits and riding techniques based on the α angle. (A) Mean (± SD) value of α angle for each of the two riding techniques per gait and (B) associated gait proportion for the two loops under interest for dyad 1. At both trot and canter gaits, the two-point technique is systematically associated with a smaller α angle. More than half of the strides are performed at the rising trot.

As it appears on Figure 4, HRC patterns clearly differed between canter and trot. Specifically, the ellipsoidal profiles observed during canter, for both the two-point and the sitting techniques indicated that the rider and the horse were globally coupled in-phase during all the cycle. A larger area of the ellipse was observed for the two-point technique, which roughly reflected a positive time-delay between the rider and horse displacements. Analysis of RP at the lower reversal point confirmed this hypothesis. Indeed, it revealed that the two-point riding technique was associated with a larger RP (29.9±8.2 deg) than the sitting technique (8.0±6.3 deg) (Figure 5). Thus, the coordination patterns adopted during the two-point technique and the sitting one seemingly differed mostly with respect to time delay between the displacements of the horse and the rider. The analysis of α angle showed also a lower value of inclination for the two-point technique relative to the sitting one, α = 47.2±2.8 deg and 57.0±1.5 deg respectively (Figure 6A).

Profiles of Lissajous plots observed during trot were clearly different from those observed during canter, and they differed also between the two adopted techniques. Specifically, the trot was characterized by two successive, but different, coordination patterns in the rising technique and by two similar (overlapping) ones in the two-point technique (Figure 4B). In rising trot, two ellipses were observed, one with an angle of 66.8±4.1 deg and the other being flat. This flat part presumably reflects freezing of the rider oscillator during one horse DV oscillation. In this ellipse, the two oscillators were decoupled whereas on the other ellipse the horse and rider presented a coupling similar to that observed in canter. During this coupled phase, the RP averaged 13.3±5.8 deg (Figure 5). In the two-point trot, the coordination pattern presented two overlapping ellipses. In this technique, the inclination of Lissajous plot was smaller (α = 37.9±8.7 deg) than in rising technique (Figure 6A). As in canter, the time delay between horse and rider's displacement was larger in two point technique (24.0±10.0 deg) than in the second technique (13.3±5.8 deg at the rising trot). This technique, which was dynamically less consistent than the rising trot (Figure 5) and the least used technique (Figure 6B), should certainly correspond to particular situations of very infrequent use.

### Analysis of the six dyads

**Figure 7:**
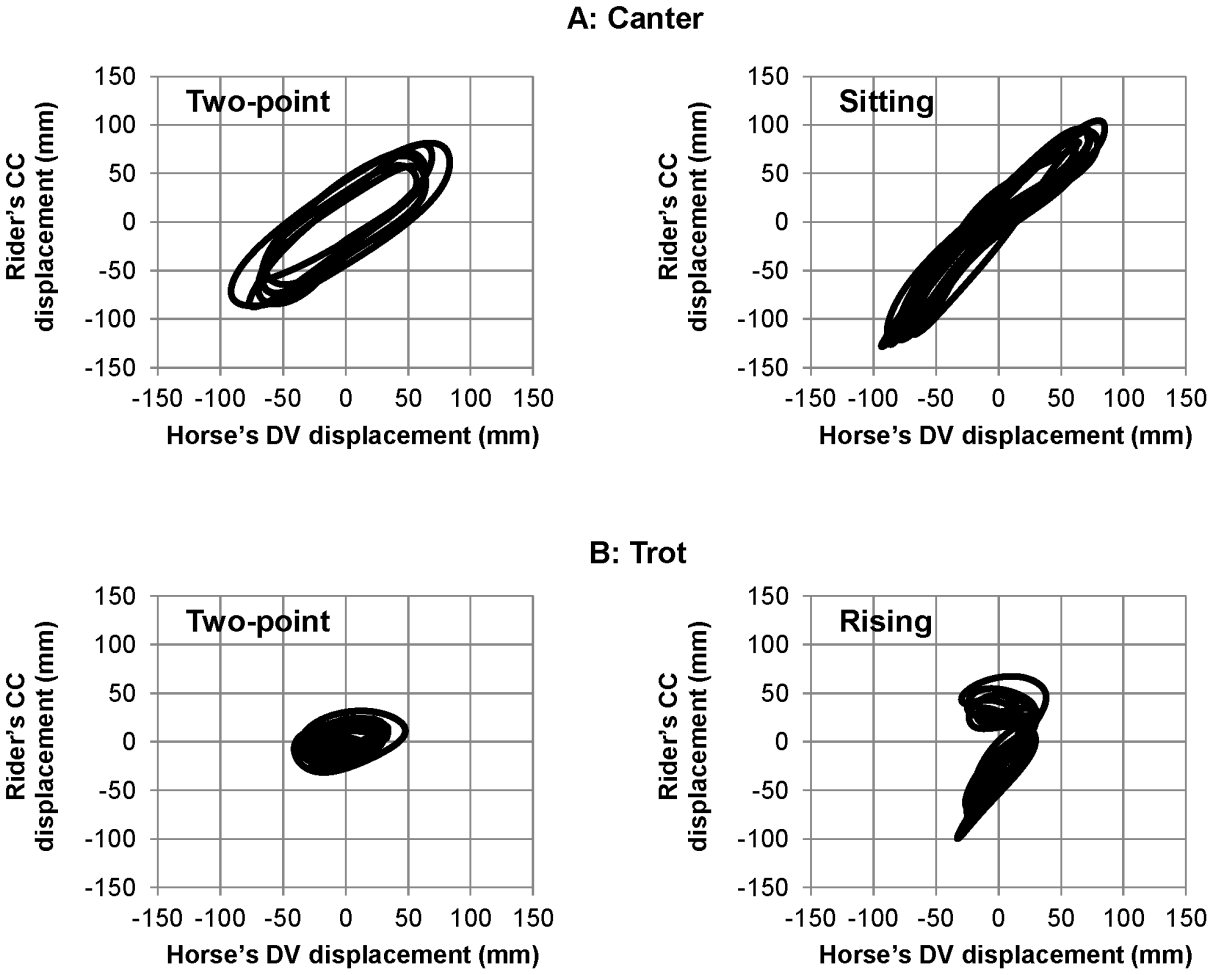
Lissajous plots characterizing each riding technique for the six dyads. Individual Lissajous plots (averaged over 100 strides) for each combination of horse's gait (panels A & B) and riding technique (left and right panels).

The individual analysis carried out for D1 was then extended to each of the five other dyads. Applying the methods used for D1 allowed easy identification of horse gait and riding technique in each dyad (see Figure 7; individual patterns are presented as supplementary material in Figures S1, S2, S3, and S4).

#### Frequency of horse gait-riding technique combinations across the different races

The mean frequencies of the different combinations observed in the different dyads that is, during the 6 different races showed large variability, thereby suggesting that the occurrence of the different patterns strongly depended on race conditions. In particular, the loop lengths varied from 20 to 42 km and race's profiles varied from flat to craggy (from ±504 m to ±3344 m in positive/negative altitude). (Table 1)

**Table 1:** Proportion of each combination of horse gait-riding technique per dyad.

Riding technique percentage (%)	Dyad 1	Dyad 2	Dyad 3	Dyad 4	Dyad 5	Dyad 6	Mean ± SD
Two-point Canter	17,9	57,1	22,5	11,8	70,8	25,1	34±24
Sitting Canter	16,0	14,9	25,4	43,5	4,8	20,6	21±10
Two-point Trot	7,6	9,2	1,5	24,3	2,8	24,3	12±17
Rising Trot	58,5	18,8	50,6	20,4	21,6	30,0	33±13

Then, statistical analyzes were carried out on the mean values of the different variables (see table 2).

**Table 2:** Group mean and SD values of the different variables in the four horse gait-riding technique combinations.

	Two-point Canter	Sitting Canter	Two-point Trot	Rising Trot
Alpha Angle (deg)	47.6±2.3	55.6±6.9	44.8±1.5	66.9±1.8
RP(deg)	15.6±7.8	−2.3±9.4	19.8±5.8	7.9±7.3
SD RP (deg)	9.5±1.6	8.4±3.6	11.1±3.4	6.1±1.2

#### Alpha angles

The analysis of α angle, showed a significant effect of gait-technique combinations on α angle (F(3,20) = 40.59, p<.001). The Newman-keuls *post-hoc* test showed that α angles were significantly higher in rising trot (66.9±1.8 deg) than in sitting canter (55.6±6.9 deg, p<.01). It was also higher than those observed in both canter and trot two-point techniques, which didn't differed between each other (47.6±2.3 deg and 44.8±1.5 deg, respectively; p>.05). Alpha angles observed in sitting canter were also higher than in the two-point (canter and trot) combinations (p<.01).

#### Mean and SD of Relative Phase

The ANOVA on the RP indicated significant differences between gait-technique combinations (F(3,20) = 9.54, p<.001). The Newman-Keuls *post-hoc* test showed that RP was significantly lower in sitting canter (−2.3±9.4 deg) than in rising trot (7.9±7.3 deg, p<.05) and in the two-point techniques (two-point canter = 15.6±7.8 deg and two-point trot = 19.8±5.8 deg, p<.05), which were not significantly different one from the other. RPs of two-point trot and canter were also larger than those observed in rising trot (p<.05).

The analysis of variability of RP indicated significant differences between the four patterns (F(3,20) = 3.75, p<.05). Variability of RP in the rising trot (6.1±1.2 deg) was smaller than in two-point trot (11.1±3.4 deg, p<.05). No significant differences were found for the other coordination patterns.

## Discussion

As a prerequisite, the present work confirmed that combination of accelerometric data recorded by two classic devices – one located on horse (Equim) and the other on rider (Locom) – allowed identification of the horse's gait patterns and riding techniques, respectively. Indeed, by a double integration of the accelerometric data, we obtained the changes in relative position along time of the horse and rider in real endurance racing condition. Thus, the present study extends the findings of those carried out in dressage [5] by characterizing horse-rider coordination patterns during a natural, endurance race situation. This was achieved using simple representations capturing the spatio-temporal relations between the horse and the rider over repeated strides that is, Lissajous plots and relative phase analysis at specific points of movement cycle. Mean and standard deviation of relative phase at the lower reversal point as well as mean orientation angle of the displacement-displacement plot (α angle) revealed the presence of different coordination patterns over the time course of the race.

### Identification of horse's gait patterns

Gait patterns were identified on the basis of the frequency of vertical oscillations of the horse. Indeed, since the trot included two oscillations per stride of the DV horse displacement and the canter only one, frequency allowed reliable identification of horse's gait patterns. With respect to the mean frequency of vertical displacements, results showed that stride frequency at the trot was slightly lower than stride frequency at the canter (1.60±0.07 Hz and 1.84±0.08 Hz, respectively). Supporting our analysis based on the vertical oscillation frequency rather than on the stride one, the range of stride frequency was smaller at the trot (1.3 to 2.0 Hz) than at the canter (1.4 to 2.7 Hz), but they were overlapping.

L2 and L3 loops were performed at similar average speeds of 5.26±0.29 and 5.13±0.63 m.s^−1^, respectively, which allowed using both trot and canter [41], [42]. This hypothesis was confirmed by the analysis of distribution frequency of trot and canter gait patterns, which showed that both patterns were used but with different averaged frequencies in each endurance race. This is not surprising since, contrary to some other competitive situations, in endurance races horses are not strictly constrained to adopt a single gait pattern. The frequency of adoption of the different gait patterns presumably reflects an on-line adaptation process to constraints of various origins (change in the nature of the track, either horse or rider's fatigue …), which must be accommodated by the horse-rider dyads at specific portions of the race loops. Accordingly, in the framework of Coordination Dynamics, horse gait pattern switching can be interpreted as the result of the interplay between the spontaneous dynamics of horse gait coordination patterns (see [41] for an illustrative example) and the directed dynamics superimposed by the rider to trigger or delay spontaneous horse gait pattern switching (see [43], [44], [45]). In the lack of additional information about the constraints arising during the different portions of race loops, it is impossible to identify which factor (or coalition of factors) triggered the transitions between horse gait patterns. On the basis of data reported in un-ridden horses, it can be considered that transitions between trot and canter should occur between 3 and 6 m.s^−1^. However, in un-ridden conditions, spontaneous transitions are partly attributed to the fact that due to speed variations, horses are facing critical metabolic [41], [46] or kinetic [47], [48] constraints that can be accommodated by changing gaits toward more comfortable, economical and/or stable patterns. In the present context, these transitions are hypothesized to reflect the directed dynamics of the horse-rider system, which result from the interplay between the spontaneous dynamics of the horse and the forcing imposed the rider in specific conditions of the race. On the one hand, the horse's adjustments may result from metabolic, kinetic and/or environmental constraints (changes in the track surface, slope and direction). On the other hand, forcing refers to rider's strategic choices (informational forcing) with respect to the compromise between speed and economy. In future work, the causal factors leading to switch between gait patterns will be identified through continuous recordings of speed and location at different portions of race loops.

### Identification of riding techniques per horse's gait

Once the horse's trot and canter gait patterns were identified, the subsequent analysis of the accelerometric data allowed the differentiation of emergent specific patterns in each dyad, corresponding to the major riding techniques used in endurance races: the two-point trot, the rising trot, the two-point canter and the sitting canter [32]. In the two-point trot and two-point canter techniques, the rider predominantly remains off the saddle that is, in equilibrium in his two stirrups. Consequently, the rider's body moves over small amplitude with respect to a word inertial frame [4]. Thus, the analysis of the maximal amplitude of the rider's craniocaudal (CC) displacement (for each stride) led to the identification of two distinct ranges of CC amplitudes per gait. Thus, within each gait, the lowest CC amplitudes were attributed to the two-point technique. It should be mentioned, however, that the overlap of the range of rider's CC displacement still required the two gait patterns to be identified at first. Comparison with previous studies confirmed the absence in endurance racing of walk gait or sitting trot technique, which are currently used in dressage [5].

This differentiation of the riding techniques revealed that the mean frequencies of the horse gait and riding technique combinations observed in the 6 dyads during the 6 different races showed large variability. These results suggested that the occurrence of the different patterns strongly depended on race conditions. Despite profiles of races varying from flat to craggy, the 6 dyads used the four patterns of coordination. To explain these results, one can hypothesize that riders needed, in addition to adapt to track constraints, to alternate static and dynamic lower limb muscle actions. By attenuating the blood flow restriction, this alternation is likely to delay the metabolic type of muscle fatigue commonly induced by sustained static muscle actions [49]. In rising trot, this alternation was occurring every half stride. In addition, the two-point technique requires to coordinate activities of lower limbs, upper limbs and trunk muscles, which presumably results in substantial additional mechanical work, a higher metabolic cost and significant changes in the cardio-respiratory response for the rider [50]. Accordingly, the use of the rising trot technique might be guided by lower metabolic and mechanical constraints applied to the rider. Similarly, Pfau [4] recently demonstrated in speed races that, for the horse, the two-point technique attenuates the acceleration and deceleration of the jockey's weight in each stride cycle. Although beneficial for the horse, the two-point riding technique remains, for the same reasons as above-mentioned for the trot, probably most metabolically and mechanically costly for the rider. By alternating the riding techniques, riders were presumably aiming to delay the onset of fatigue while searching for better performance.

### Horse-Rider coordination patterns

During the different associations between gait and technique, the horse-rider coupled system produced complex coordination patterns. These patterns can be distinguished on the basis of the analysis of relative phase and relative amplitude of horse and rider vertical displacements over the stride cycles (i.e. alpha angles).

A first observation is that the averaging of the numerous cycles recorded over the long duration of the different loops resulted in very reproducible patterns across the six dyads (Figure 7). Thus despite changes in environmental conditions within and between races, the coalition of constraints was strong enough to shape stable patterns of interactions between the two components of the horse-rider system. The stability of the different patterns was attested by variability analysis, which showed lower values of SD of relative phase, when compared to those observed in classic laboratory studies on interlimb coordination (e.g., [51]) or even on interpersonal coordination [24]. Thus, even when measured in natural situations and over a very large number of strides, constraints led to very stable horse-rider coordination patterns, at least at lower reversal point of the gait cycle.

Lissajous figures captured the four specific spatio-temporal patterns of horse-rider coordination adopted by the different dyads. They showed that the patterns produced at the canter and trot clearly differed from each other. At the canter, ellipsoidal figures inclined to the right, indicated that each dyad, horse and rider were tightly coupled in phase. However, the difference between α angles associated with the two ellipses showed that spatial coupling was stronger during the two-point technique than during the sitting canter. Indeed, in the two-point technique, α angle was closer to 45° than during sitting, which showed the amplitude of displacements of both components of the horse-rider dyad to be closely related. One can speculate that the multi-articulated rider system allowed lower limbs to be presumably functional in this respect. By acting as damping oscillators, they permitted the riders to adjust their movements to the horse's oscillations. In the sitting canter technique, mean α angle was significantly higher than 45° (55°), thereby showing that rider's displacements were larger than horse's displacements. This result suggests that “sitting” should not be taken in its usual sense in the present situation. Since the amplitude of the vertical displacements of the riders was larger than those of the horse, the riders did not remain in the saddle. Instead, they only touched it during a brief portion of the cycle, which permitted them to propel themselves above the saddle at the maximal amplitude of horse's upward oscillation (see upper extremity right of the Lissajous plot on Figure 4 and Figure 7). Then, the riders moved down and synchronized their displacements with those of the horse at the reversal point of the downward oscillation and so on.

The patterns observed during canter were quite different from those observed during trot. Indeed, both presented a flat orientation suggesting a freezing of rider's displacements during about a half cycle (rising trot) and during almost all the cycle (two-point technique). In particular, the pattern corresponding to the rising trot technique presented a remarkable 7-like shape type, which presumably resulted from the fact that for half a stride the riders remained in close contact with the saddle, whereas during the subsequent half-stride they were in equilibrium on the stirrups. This was associated with larger alpha angles, meaning that for the portion during which the horse and the rider were coupled in-phase, the displacements of the rider were larger than those of the horse. Then, the riders remained in the higher “rising” position. By freezing their own vertical oscillation during half a stride (horizontal part of the 7), the riders were thus modifying their own coordination within the trot cycle. Therefore, such freezing of the “rider oscillator” allowed the adoption of a 1∶1 coordination (one oscillation for one stride) although the “horse oscillator” has twice the rider's frequency. The freezing phenomenon was also observed, though less marked, in the two-point trot technique. Indeed, the flat portion of the corresponding Lissajous figure indicated that the riders probably attempted to freeze their oscillations during the entire stride but still oscillated slightly, thereby failing to fully decouple their displacements from those of the horse during the two half strides. It is noticeable that at the trot, a third mode of coordination (sitting trot) could have occurred if the riders would have remained all the time sited in the saddle. In this case, the observed pattern would be two identical trajectories, similar to the inclined part of rising trot, in the Lissajous figure. Our data showed, however, that the riders of the different dyads never adopted this solution, which is rather used in dressage [5].

Another important result of the present study lies in the analysis of relative phase (RP) between horse and rider displacements along the vertical axis, measured at lower reversal point, for the different dyads. On the one hand, the RP values are found to be mainly dependent on the riding technique. Specifically, the RP calculated for the rising trot and sitting canter techniques were significantly lower than those observed in the two-point technique at the trot and canter gaits, which are close to each other. Lower values observed in the sitting and rising trot presumably reflected the fact that, in these two techniques, riders were in close contact with the saddle during a part of the cycle, especially at the lower reversal point. In support of this hypothesis, it is noticeable that variability of relative phase at lower reversal point was smaller for sitting canter and rising trot patterns than for the two others. These results suggest that the rider's technique was more constraining on the type and stability of the adopted coordination pattern than was the horse gait. This gives support to the existence of a specific coordination within the horse-rider dyad that would not depend on the horse's gait. These results can be compared with those observed by Wolfram et al. [5]. In this study, analysis of continuous RP (averaged over several full cycles), showed more variability during sitting canter than during rising trot or sitting trot. The authors suggested that larger variability could be explained by the use of rider suspension phase to resynchronize with horse's oscillation. In the present study, RP was analyzed at the lower reversal point and showed that mean and variability of RP values were close in sitting canter and rising trot (8.0±6.3 and 13.3±5.8 deg, respectively). The stability of the coordination pattern observed during sitting canter and rising trot presumably results from the anchoring provided by haptic contact information of the rider with the saddle. This observation points out the importance of analyzing discrete relative phase at the lower reversal point of the cycle. Indeed, lower reversal portion appears to be very functional relative to the upper portion, which corresponds to a freezing of rider's displacement in rising trot. The larger RP values found in the two-point trot and canter techniques may be attributed to the fact that the coordination pattern is less stabilized in the two-point technique due to the lack of informational contact with the saddle than in the rising trot and sitting canter.

Thus, whereas phase synchronization is considered spontaneous in most weakly coupled oscillator systems, the present results suggest that they are open to stabilization by haptic informational exchange. It is certainly the case that rider's expertise manifests through sensitivity to and anticipation of the horse's motion. Several studies have shown that expertise leads to lower variability on horse [27], on rider [52], [53], [54] or on HRC [2], [3]. In our case the rider being expert, the variability of the relative phase is very low, which reflects the stability of coordination patterns adopted even over long time duration of endurance race.

## Conclusion and Perspectives

This study had three main objectives. First, it aimed to validate a measurement system combining the relative horse and rider movements recorded by two 3D accelerometers in natural (i.e. complex) situation of endurance race competition. The second objective was to describe and quantify horse's gait and riding techniques most frequently used, on the basis of behavioral patterns identified in expert horse-rider dyads in a natural competitive situation. The third objective was to characterize the coordination patterns exhibited by the horse-rider system as emergent patterns in a complex self-organized dynamical system. In this respect, the interest and originality of the present study is to demonstrate that, even in the lack of detailed information about the racing constraints, one can characterize emerging patterns. These patterns might be then taken as basis to further study the detailed conditions of their emergence and dynamics. In particular, it would be of interest to determine whether changes are triggered by environmental-, rider- (e.g. fatigue in lower limb muscles) or horse-related constraints or even a weighted combination of them. Studies are in process in our group to explore these issues. The natural and competitive situation of endurance race clearly differs from the commonly studied un-ridden situations, in particular those performed on a treadmill. In addition, the present study is quite complementary to those recently performed in natural conditions of speed races [4], which used similar technologies to study the horse-rider coupling, but in a single riding technique.

A limit of the present study resides in the small number of horse-rider dyads. Accordingly, further investigations including dyads of different level of expertise should be planned to examine how coordination patterns stabilize and change over the long-term learning process, which does not only involve changes in individual behavior of the horse and the rider, but also improvement of their cooperation.

## Supporting Information

Figure S1 **Lissajous plots of Horse-rider coupling (HRC) at two-point canter for the six dyads.** Each panel combines the horse's dorsoventral (DV) displacement (horizontal axis) with the rider's craniocaudal (CC) one (vertical axis) for each dyads at two-point canter. Emphasizing the stability of the HRC patterns, the mean Lissajous plot (black curve) is very similar to the plots of 100 successive strides (grey curves).
(TIFF)

Figure S2 **Lissajous plots of Horse-rider coupling (HRC) at sitting canter for the six dyads.** Each panel combines the horse's dorsoventral (DV) displacement (horizontal axis) with the rider's craniocaudal (CC) one (vertical axis) for each dyads at two-point canter. Emphasizing the stability of the HRC patterns, the mean Lissajous plot (black curve) is very similar to the plots of 100 successive strides (grey curves).
(TIFF)

Figure S3 **Lissajous plots of Horse-rider coupling (HRC) in the two-point trot for the six dyads.** Each panel combines the horse's dorsoventral (DV) displacement (horizontal axis) with the rider's craniocaudal (CC) one (vertical axis) for each dyads at two-point canter. Emphasizing the stability of the HRC patterns, the mean Lissajous plot (black curve) is very similar to the plots of 100 successive strides (grey curves).
(TIFF)

Figure S4 **Lissajous plots of Horse-rider coupling (HRC) at Rising trot for the six dyads.** Each panel combines the horse's dorsoventral (DV) displacement (horizontal axis) with the rider's craniocaudal (CC) one (vertical axis) for each dyads at two-point canter. Emphasizing the stability of the HRC patterns, the mean Lissajous plot (black curve) is very similar to the plots of 100 successive strides (grey curves).
(TIFF)
