# Supplementary figures and images for: Patterns of Horse-Rider Coordination during Endurance Race: A Dynamical System Approach
Source: PLoS One. 2013 Aug 5;8(8):e71804. doi: 10.1371/journal.pone.0071804 (PMC3733789; doi:10.1371/journal.pone.0071804)

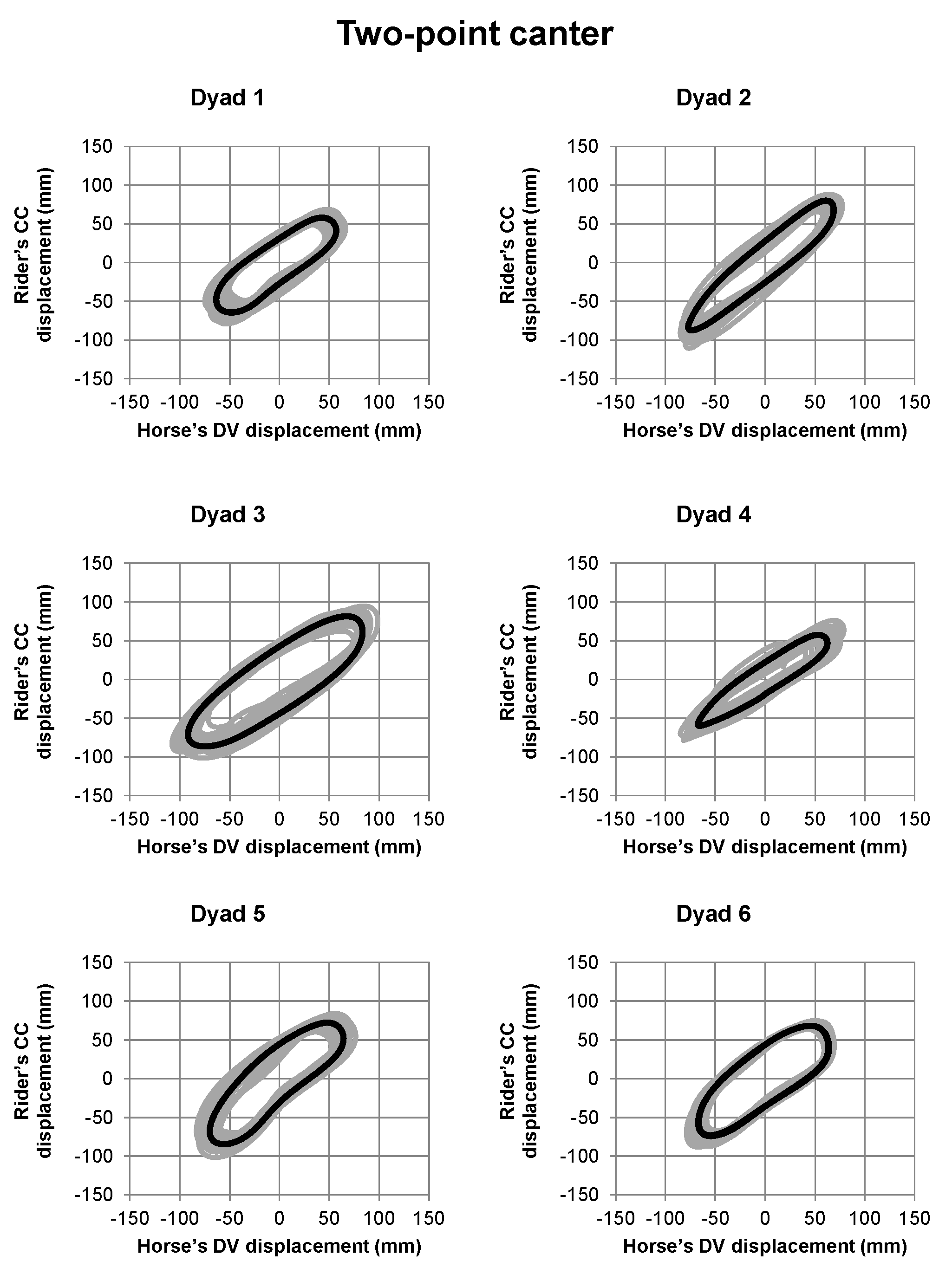

Supplement: Figure S1 — Lissajous plots of Horse-rider coupling (HRC) at two-point canter for the six dyads. Each panel combines the horse's dorsoventral (DV) displacement (horizontal axis) with the rider's craniocaudal (CC) one (vertical axis) for each dyads at two-point canter. Emphasizing the stability of the HRC patterns, the mean Lissajous plot (black curve) is very similar to the plots of 100 successive strides (grey curves). (TIFF) [file pone.0071804.s001.tiff]

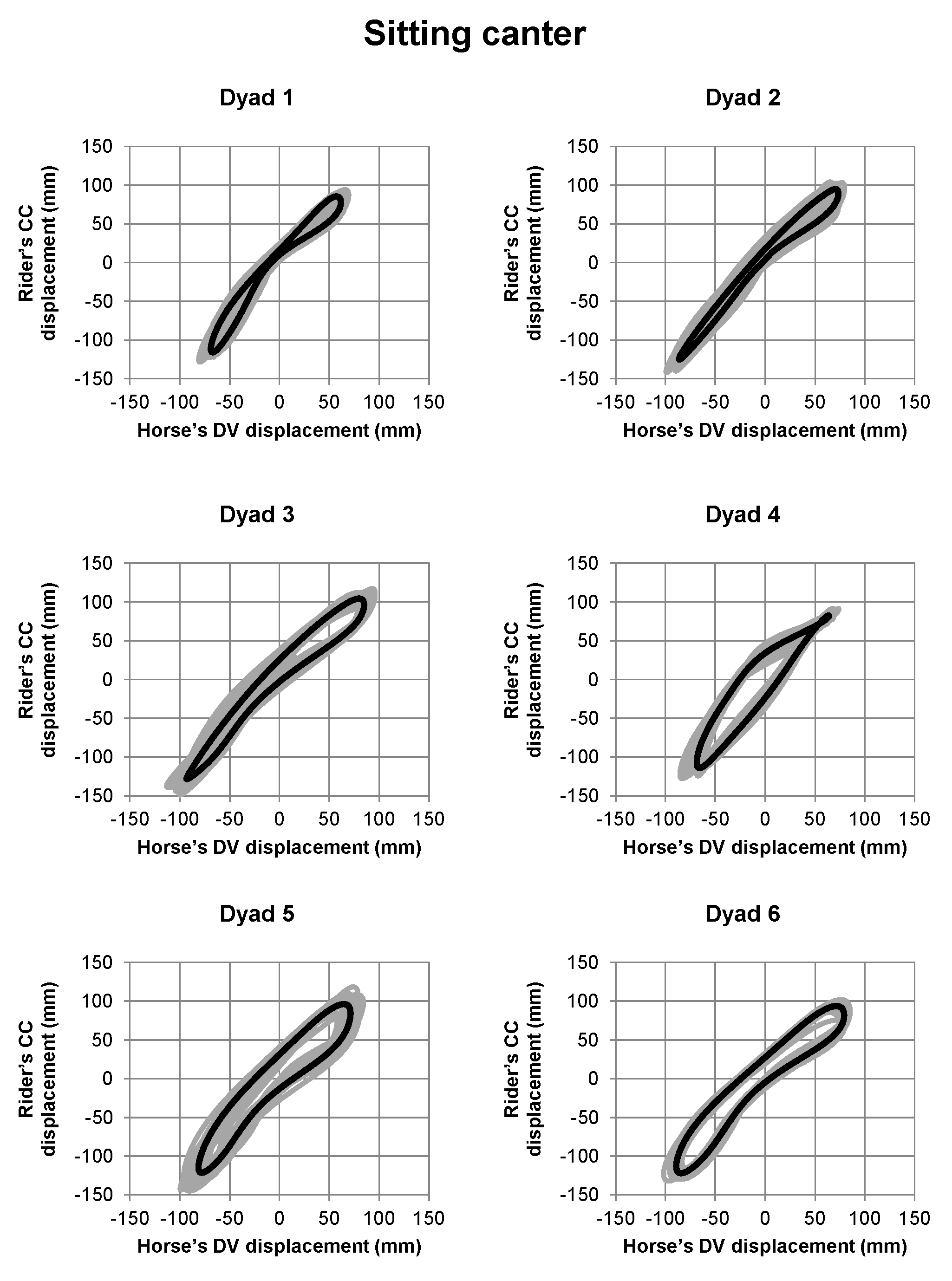

Supplement: Figure S2 — Lissajous plots of Horse-rider coupling (HRC) at sitting canter for the six dyads. Each panel combines the horse's dorsoventral (DV) displacement (horizontal axis) with the rider's craniocaudal (CC) one (vertical axis) for each dyads at two-point canter. Emphasizing the stability of the HRC patterns, the mean Lissajous plot (black curve) is very similar to the plots of 100 successive strides (grey curves). (TIFF) [file pone.0071804.s002.tiff]

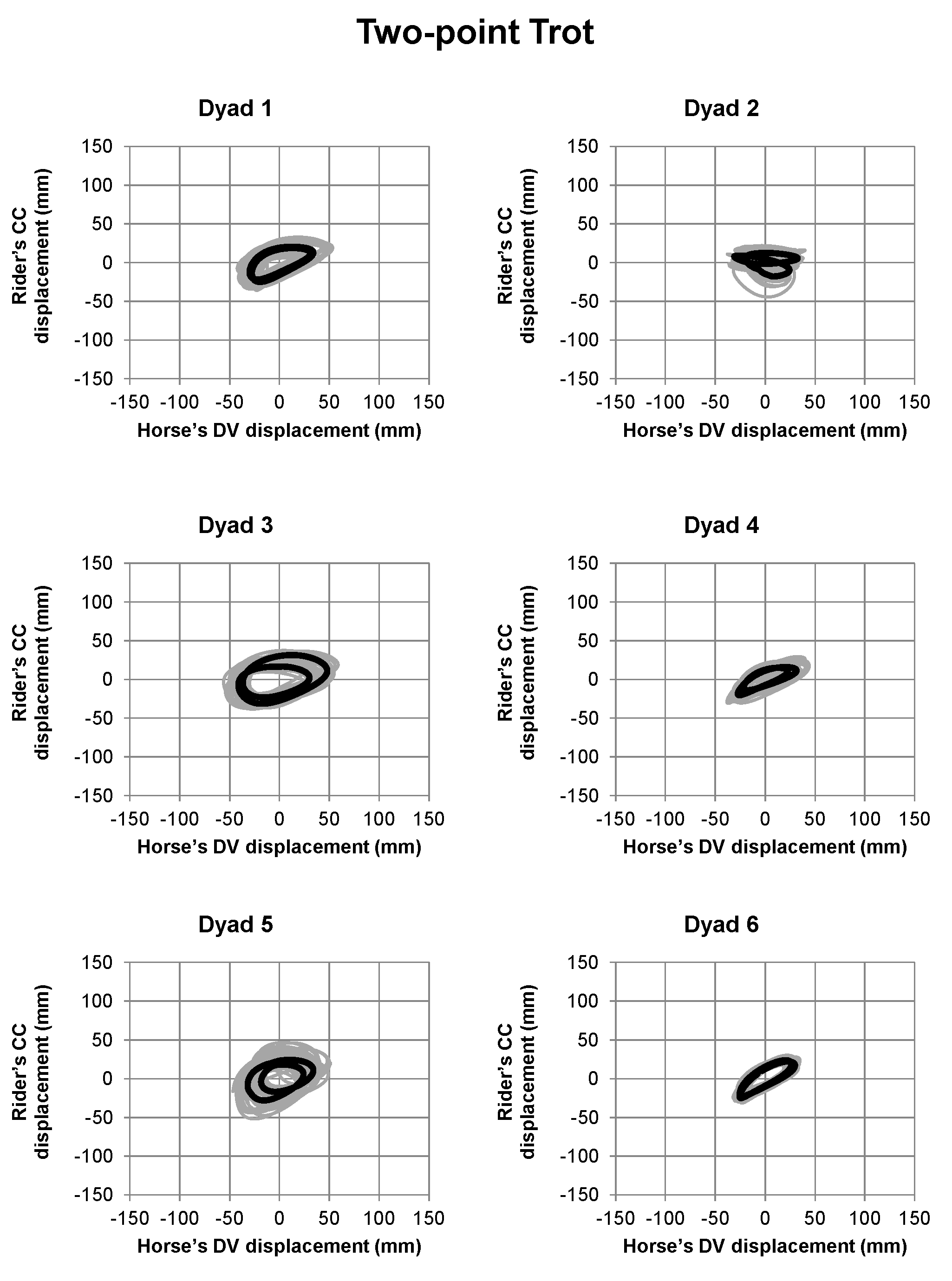

Supplement: Figure S3 — Lissajous plots of Horse-rider coupling (HRC) in the two-point trot for the six dyads. Each panel combines the horse's dorsoventral (DV) displacement (horizontal axis) with the rider's craniocaudal (CC) one (vertical axis) for each dyads at two-point canter. Emphasizing the stability of the HRC patterns, the mean Lissajous plot (black curve) is very similar to the plots of 100 successive strides (grey curves). (TIFF) [file pone.0071804.s003.tiff]

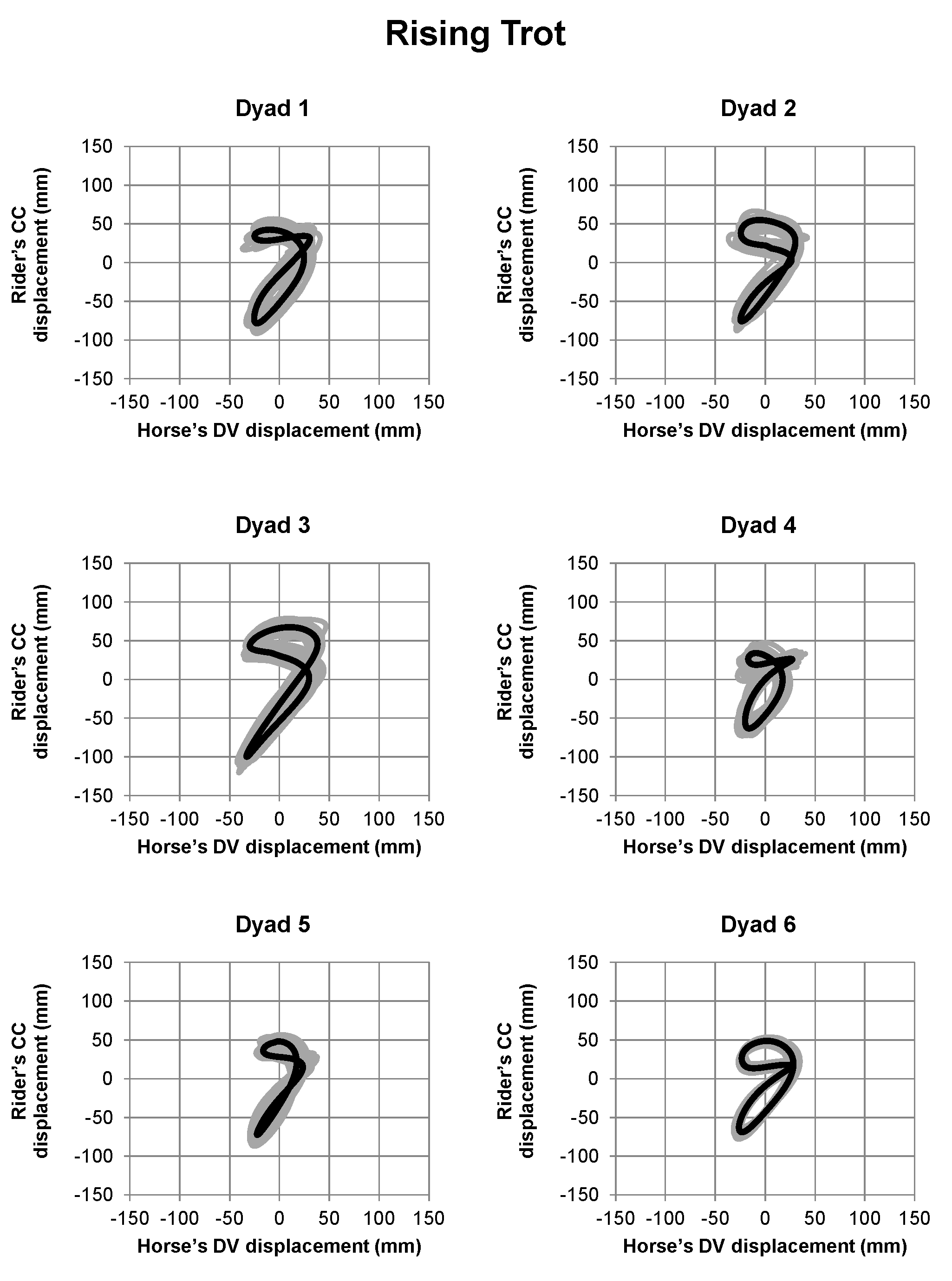

Supplement: Figure S4 — Lissajous plots of Horse-rider coupling (HRC) at Rising trot for the six dyads. Each panel combines the horse's dorsoventral (DV) displacement (horizontal axis) with the rider's craniocaudal (CC) one (vertical axis) for each dyads at two-point canter. Emphasizing the stability of the HRC patterns, the mean Lissajous plot (black curve) is very similar to the plots of 100 successive strides (grey curves). (TIFF) [file pone.0071804.s004.tiff]
